# Jet-Breaking Extrusion of Alginate–Chitosan Capsules for Encapsulation of Plant Growth–Promoting Extremophilic Fungi

**DOI:** 10.3390/microorganisms13051123

**Published:** 2025-05-14

**Authors:** César Arriagada-Escamilla, Javier Ortiz, Nicole Iturra, Javiera Soto, Eduardo Morales

**Affiliations:** 1 Laboratorio de Biorremediación, Departamento de Ciencias Forestales, Facultad de Ciencias Agropecuarias y Medioambiente, Universidad de La Frontera, Av. Francisco Salazar 01145, Temuco 4811230, Chile; javier.ortiz@ufrontera.cl (J.O.); n.iturra03@ufromail.cl (N.I.); javiera.soto@ufrontera.cl (J.S.); 2 Scientific and Technological Bioresource Nucleus (BIOREN), Universidad de La Frontera, Temuco 4811230, Chile; eduardo.morales@ufrontera.cl

**Keywords:** alginate, chitosan, capsules, extremophilic root-associated fungi, ionic gelation, jet breaking, extrusion

## Abstract

Drought and metal pollution severely impact plant growth. Root-associated extremophilic fungi can improve plant performance, and their encapsulation improves protection and effectiveness. This study optimized the encapsulation conditions for an extremophilic fungus with plant growth-promoting traits using alginate–chitosan capsules. An endophytic fungus was isolated from the roots of *Neltuma chilensis* from the Atacama Desert and identified via internal transcribed spacer (ITS) sequencing. Its plant growth-promoting traits, including exopolysaccharide, ammonium, siderophore, and indole acetic acid production and phosphorus solubilization, were evaluated. Freeze-dried *Penicillium nalgiovense* was encapsulated using jet-breaking extrusion, and capsule morphology and fungal survival were assessed via scanning electron microscope (SEM), confocal laser scanning microscopy (CLSM), and viability tests. Using Taguchi’s design, optimal conditions for sphericity (0.914 ± 0.002) and mean size (3.232 ± 0.087 mm) were achieved with 1% chitosan, a 5 cm distance to the gelation bath, and a 40 Hz vibration frequency. CLSM analysis confirmed the presence of the chitosan outer layer, revealing the capsule’s coating material encapsulating the fungus *P. nalgiovense*. The encapsulated fungus remained viable across disinfection times, demonstrating effective protection and gradual release. These findings emphasize the need for precise parameter control in fungal encapsulation, providing a basis for developing robust bioinoculants to support plant resilience in extreme environments.

## 1. Introduction

Drought and metal pollution are severe environmental problems affecting plant establishment and crop productivity worldwide. Both abiotic stresses are directly related to anthropogenic intervention and the overexploitation of natural resources. Plants are one of the living organisms most affected by both climatic alterations and contamination of the soil solution [1]. These stressed environmental conditions are not new to native ecosystems, and several plants and microorganisms have developed genetically evolved adaptation mechanisms to grow in harmful environments such as high-altitude slopes in the Andes or the Atacama Desert [2]. Here, plants can establish specific symbioses with microorganisms living in the rhizosphere to improve their growth and development. Some microbial taxa can thrive in extreme environmental conditions such as salinity, high soil surface temperature, acidic soils, high amounts of metals, and long dry seasons. These microorganisms are known as extremophilic root-associated fungi and represent an exciting opportunity to improve plant performance in the current climate change scenario [3]. In addition, inoculation of root-associated fungi can reduce water and fertilization requirements and restore leaf hydration in drought conditions, making their production more profitable [4]. For field applications, encapsulation procedures have been shown to preserve the ability of fungi to develop hyphae, especially under stressful environmental conditions [4,5]. The encapsulation of microorganisms involves enclosing or entrapping these microorganisms within a protective barrier. This encapsulation enhances the transfer of nutrients and substrates between the interior of the capsule and the surrounding environment, thereby promoting more efficient interactions and growth [6]. The primary advantages of encapsulation include enhanced protection against adverse environmental conditions and competition with native microorganisms, precise control over the timing of release, improved utility, preservation of strain purity, maintenance of genetic stability, and more convenient storage [7,8]. During encapsulation, a new structure consists of two distinct components: the core, which contains the microorganism suspension, and the wall or shell, composed of the encapsulating material. The resulting particles are classified according to their internal structure. Particles with a hollow core and a heterogeneous structure, where the wall surrounds the core, are called capsules. In contrast, particles with a solid interior or those in which the core is homogeneously dispersed within the wall, either dissolved or suspended, are known as beads [9]. However, to our knowledge, root-associated fungal capsules have not been designed as alternatives to beads.

Currently, several techniques are employed for microorganism encapsulation, including ionic gelation, spray drying, lyophilization, fluidized bed, extrusion, electrospinning, liposomes, and complex coacervation [10]. One of the most common methods for encapsulating microorganisms is ionic gelation, in which microorganisms are incorporated into alginate gel beads by dropping a soluble sodium alginate solution into an aqueous Ca^2+^ solution, typically containing calcium chloride [11]. Nevertheless, this study introduces a novel strategy that combines ionic gelation with jet-breaking extrusion technology to enable the encapsulation of extremophilic fungi at high fungal loads. This innovative approach produces uniform, spherical capsules by generating controlled droplets under an electric field, thereby enhancing encapsulation efficiency, protection, and applicability [12]. The method is simple, cost-effective, and suitable for producing fungal-loaded capsules, positioning it as a promising alternative for the development of bioinoculants [13].

In recent years, there has been growing interest in using capsules or beads derived from natural polymers due to their renewability, biodegradability, biocompatibility, and non-toxicity [14]. For instance, incorporating additional natural polymers, like chitosan, in combination with alginate, can modulate the physicochemical properties of the final matrix, thereby enhancing the encapsulation process [14,15]. The primary function of calcium alginate gel is to trap the material to be encapsulated quickly and under mild conditions, forming spherical particles. These particles can then bind to chitosan, creating a robust membrane complex that stabilizes the ionic network of the gel while reducing and controlling its permeability [16]. In this context, chitosan has gained significant attention as a promising candidate for encapsulation [17]. This biopolymer is highly versatile due to its biological properties, which include non-toxicity, biocompatibility, biodegradability, hemostatic activity, and both antibacterial and antifungal properties [18,19]. The positively charged amino group of chitosan can interact electrostatically with the negatively charged groups of sodium alginate to form composite particles through intermolecular interactions or even a linked polymeric network [20].

The development of innovative encapsulation systems necessitates the optimization of various parameters to achieve stability and effectiveness [21]. Key characteristics such as sphericity and size are influenced by multiple factors, including nozzle size, the concentration and viscosity of gelling solutions, vibration frequency, and the distance between the nozzle and the gelling bath [12,14]. This study employs the Taguchi method to optimize the process conditions for preparing alginate–chitosan capsules used to encapsulate extremophilic root-associated fungi. Taguchi’s method for experimental design is a streamlined statistical approach that employs predefined matrix arrangements to efficiently investigate the influence of multiple variables. The use of orthogonal arrays allows for the precise estimation of individual factor effects while significantly minimizing the number of experimental runs. This methodology has proven effective in identifying key design parameters and determining their optimal settings through empirical testing [22]. Our previous experimental work has validated the use of the Taguchi method as an effective approach for optimizing encapsulation processes [21,23]. According to the above considerations, the aim of this study was to evaluate the process conditions on the sphericity of alginate–chitosan capsules containing extremophilic root-associated fungi with plant-promoting capabilities. In this study, the process conditions for preparing spherical alginate–chitosan capsules were determined by the Taguchi methodology with an orthogonal array L_4_(2^3^). The effect of the variables applied vibration frequency (frequency: 40–120 Hz), distance to the gelation bath (distance: 5–15 cm), and concentration of the encapsulating material (chitosan: 1–2%) on the capsules’ sphericity was evaluated. In addition, particle size, morphology, and survival of encapsulated fungi were investigated.

## 2. Materials and Methods

### 2.1. Chemicals

All chemical reagents used in this study were of analytical grade and employed without further purification. Sodium alginate and chitosan were purchased from Sigma-Aldrich (St. Louis, MO, USA), while calcium chloride dihydrate (CaCl_2_·2H_2_O) and glacial acetic acid were obtained from Merck (KGaA, Darmstadt, Germany). All other reagents were also of high purity and analytical grade.

### 2.2. Isolation and Molecular Identification of Extremophilic Endophyte Root-Associated Fungus

The endophyte fungus was isolated from roots of *Neltuma chilensis* C.E. Hughes & G.P. Lewis, trees naturally subjected to abiotic stress conditions including drought, salinity, and metal stress ecosystem in the hyperarid Atacama Desert, North Chile (22°58′10″ S, 68°09′53″ W). Root samples were collected from twenty plants and thoroughly washed under running tap water to remove rhizosphere soil. The cleaned roots were then transferred to 50 mL Falcon tubes and surface-sterilized in a laminar flow cabinet using 0.5% sodium hypochlorite, followed by 100% ethanol. Subsequently, the roots were rinsed thoroughly with sterile deionized water. An aliquot of the final rinse was plated on Luria–Bertani agar and potato dextrose agar (PDA) to confirm the absence of rhizospheric microorganisms on the root surface. Finally, root segments were placed on Petri dishes containing malt extract agar, PDA, oatmeal agar, and water agar. Individual fungal strains were purified on PDA and stored on separate plates at 4 °C, with periodic subculturing to maintain viability.

For molecular identification, the fungal strain was cultured in potato dextrose broth (PDB) with continuous stirring at 26° C for 7 days, and genomic DNA was extracted using the DNeasy plant Pro kit (Qiagen ^®^, Hilden, Germany). The internal transcribed spacer (ITS) region of rDNA was amplified using universal ITS-1 (5′-TCCGTAGGTGAACCTGCGG-3′) and ITS-4 (5′-TCCTCCGCTTATTGATATGC-3′), following White et al. [24]. The obtained sequences were compared with the GenBank database using the Blast tool (http://blast.ncbi.nlm.nih.gov, accessed on 1 April 2025).

### 2.3. Characterization of Extremophilic Root-Associated Fungus

Fungus characterization was performed by screening for plant growth-promoting (PGP) traits and hydrolytic enzymes using standard procedures. The assays evaluated the production of ammonia, indole acetic acid (IAA), and siderophore, as well as the capacity to solubilize different sources of phosphorus following Ortiz et al. [25]. The 1-aminocyclopropane-1-carboxylic acid (ACC) deaminase activity was measured according to Viterbo et al. [26], while exopolysaccharide (EPS) production was determined using the method described in Soto et al. [27]. The production of protease, lipase, and cellulase activities was assessed following the methods described by Zilda et al. [28], Kasana et al. [29], and Kumar et al. [30], respectively. All assays were incubated in the dark at 26 °C for seven days. An enzyme index equal to or greater than 2.0 was considered indicative of efficient extracellular enzyme production, as described by Fuentes-Quiroz et al. [31].

### 2.4. Preparation of Encapsulating and Core Solutions

The encapsulating solution was prepared by dissolving chitosan (1 and 2% *w*/*w*) in calcium chloride solution (130 mM) and 0.5% acetic acid, with magnetic stirring (500 rpm) at room temperature until completely dissolved [32]. The core solution was prepared by dissolving sodium alginate (2% *w*/*w*) in distilled water at 500 rpm for 3 h at room temperature. Subsequently, the sodium alginate solution and the lyophilized extremophilic fungi (0.5% *w*/*w*) were mixed constantly at 500 rpm during encapsulation to avoid precipitation of the fungus.

### 2.5. Preparation of Fungi Alginate–Chitosan Capsules by Jet Breaking Extrusion

The alginate–chitosan capsules were prepared using a B-390 encapsulator (Buchi, Switzerland), according to Bennacef et al. [12], with some modifications. The alginate–chitosan solution was extruded using a nozzle with an internal diameter of 1000 µm over a beaker under constant stirring, containing the chitosan solution with calcium chloride (100 mL) as a gelling bath. During the encapsulation process, an electric charge of 2000 V and a vibration frequency were applied for the separation and dispersion of the droplets. Finally, the capsules were formed in contact with the gelling bath and hardened for 30 min. After curing, the alginate–chitosan capsules were removed from the solution by simple filtration with a sieve of appropriate mesh size. A Taguchi experimental design was then applied to prepare high-sphericity alginate–chitosan capsules. An L_4_(2^3^) matrix with three independent variables and two working levels was used, applying the criterion ‘the higher, the better’: applied vibration frequency (frequency: 40–120 Hz), nozzle-bath distance (distance: 5–15 cm), and concentration of the encapsulating material (chitosan: 1–2%), using Minitab 19 software (Minitab LLC, State College, PA, USA). The optimized theoretical equation (OTE) was determined by considering the average of the highest impact response, identifying the most important variables, and the level of work on the sphericity of the capsules. Table 1 shows the orthogonal matrix used with the design variables. The OTE was calculated according to Morales et al. [21].

OTE = T + [WL: Frequency − T] + [WL: Distance − T] + [WL: Chitosan − T](1)

where T is the total average of responses of the experimental runs, and WL corresponds to the working level of each variable in the equation.

### 2.6. Sphericity and Mean Size of Capsules

The sphericity and mean size of capsules were evaluated from images acquired by a digital camera and analyzed by ImageJ software (v. 1.46, National Institutes of Health, Bethesda, MD, USA). Approximately 15 capsules were measured to determine the following parameters:

Sphericity = (Dmax − Dmin)/(Dmax + Dmin) (2)

Mean size (Msize) = (Dmax + Dmin)/2(3)

where Dmax is the maximum diameter of the capsules, and Dmin is the perpendicular diameter of Dmax. Sphericity and mean capsule size were measured following the methodology described by Morales et al. [32].

### 2.7. Morphology

The structure or surface morphology of alginate–chitosan capsules containing fungi was analyzed using magnifications of 250× and 500× under low vacuum conditions using a SU3500 Hitachi scanning electron microscope (SEM) (Hitachi, Tokyo, Japan). Then, the alginate–chitosan capsules were analyzed by confocal laser scanning microscopy (CLSM), using Olympus FV10-ASW 1.7 software (Olympus, Tokyo, Japan), to study the distribution of chitosan as an encapsulating material. The excitation wavelength was 488 nm, and the emission wavelength was 533 nm. For this purpose, capsules (250 mg) were added to 25 mL of acetic acid (2%) and 10 mg of fluorescein isothiocyanate (FITC) to 1 mL of ethanol. Both solutions were mixed with 250 mg of capsules under stirring for 30 min to ensure molecular binding of the marker to the chitosan.

### 2.8. Survival of Encapsulated Fungus

The viability of the encapsulated fungus was evaluated based on its growth in both PDA and PDB media. To prepare the samples, 0.1 g of capsules were previously disinfected in a 1% sodium hypochlorite solution (prepared with sterile distilled water) for various times (2, 5, 10, and 20 min). After disinfection, the capsules were ground in a sterile mortar with sterile phosphate-buffered saline to extract the fungus. A 50 µL aliquot of the resulting solution was plated on Petri dishes containing PDA media and inoculated into PDB flasks. The plates and flasks were incubated at 26 °C for 7 days, and fungal growth was assessed by visual observation.

### 2.9. Statistical Analysis

The results presented are the average and standard deviation calculated from these replicate measurements. A two-way analysis of variance (ANOVA) was carried out with a significance level set at 0.05. Duncan’s test was performed in the case of significant differences detected with the ANOVA. Statistical analyses were carried out using StatSoft Inc. (2004) STATISTICA 8.0 software (StatSoft Inc., Tulsa, OK, USA).

## 3. Results

### 3.1. Isolation and Screening of PGP Traits and Hydrolytic Enzyme Activities

An endophytic root-associated fungus was isolated from the roots of *Neltuma chilensis* and identified as *Penicillium nalgiovense* (MH856385.1) through ITS sequencing. The fungus exhibited several plant growth-promoting (PGP) traits (Table 2), including high exopolysaccharide (EPS) production (102.02 mg L^−1^) and ammonium production (40.47 µmol mL^−1^). Phosphorus solubilization varied depending on the source, with the highest phosphorus solubilization index (PSI) for aluminum (5.5) and iron (5.0), while lower PSI values were observed for phytic acid (2.2) and calcium-based sources (2.6). Additionally, the fungus produced siderophores (6.13% siderophore units) and indole-3-acetic acid (IAA) at lower concentrations (0.94 µmol mL^−1^). The hydrolytic enzyme assays revealed that the fungal isolate was capable of synthesizing all the hydrolytic enzymes evaluated. Specifically, the isolate exhibited enzyme production indices of 3.4 ± 0.09 for protease, 2.1 ± 0.11 for lipase, and 3.6 ± 0.23 for cellulase.

### 3.2. Formulation of Alginate–Chitosan Capsules Containing P. nalgiovense

To improve reproducibility and ensure the formation of uniform capsules containing fungi, a more precise approach combining ionic gelation and annular jet-breaking extrusion was developed. A preliminary study was conducted to determine the main factors influencing capsule formation [32]. The selected variables included vibration frequency (40 and 120 Hz), nozzle-to-bath distance (5 and 15 cm), and chitosan concentration (1% and 2%). Outside these levels, irregular or amorphous particles were observed, confirming that non-spherical formations should be discarded.

**Figure 1:**
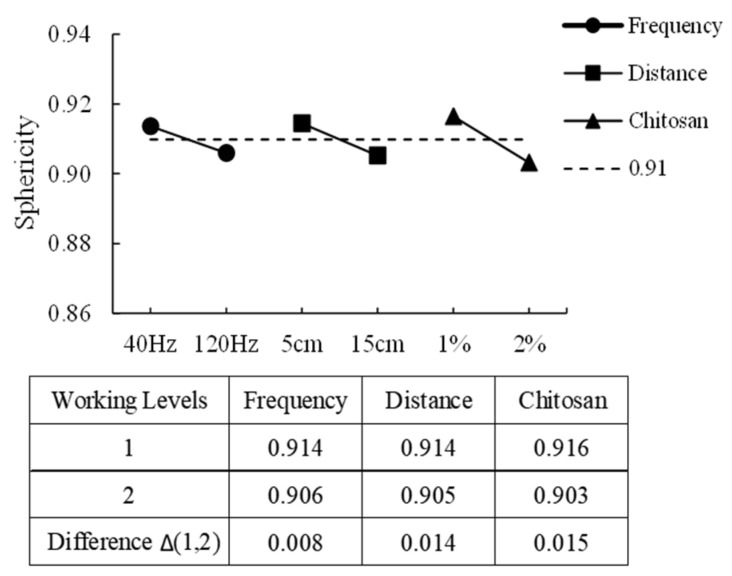
Effect of working levels of each variable in the alginate–chitosan–*P. nalgiovense* capsules on the sphericity. The greater the difference between the work levels, the greater the magnitude of the change in sphericity.

The optimization process followed the Taguchi method, using sphericity as the primary response variable. The sphericity values ranged from 0.902 to 0.925, while capsule sizes varied between 2.17 mm and 4.91 mm. The most significant factor affecting sphericity was the chitosan concentration, with an optimal value of 1%, showing a 0.015-unit difference between tested levels. The second most influential factor was nozzle-to-bath distance, with the highest sphericity (0.914 ± 0.002) observed at a 5 cm distance. Finally, vibration frequency had the least impact, contributing to only a 0.008-unit difference in sphericity. Figure 1 shows the degree of steepness of the slope as a response to the sphericity of alginate–chitosan–*P. nalgiovense* capsules, indicating that the greater the difference between the working levels for a variable, the greater the magnitude of the change in sphericity.

ANOVA analysis showed that chitosan content, distance, and frequency had determination coefficients (R^2^) of 52.8%, 25.6%, and 12.1%, respectively. The combined R^2^ value exceeded 96%, indicating a strong correlation between these process variables and sphericity. Based on these findings, the optimal process conditions for encapsulating *P. nalgiovense* were identified as 1% chitosan content, a 5 cm nozzle-to-bath distance, and a vibration frequency of 40 Hz. Under these conditions, the experimental sphericity value was 0.914 ± 0.002, with a mean capsule size of 3.232 ± 0.087 mm. Morales et al. [32] also reported that formulation and processing conditions affected the size, sphericity, and dissolution of chitosan-coated hydrogel microspheres, ranging from 3 to 5 mm and sphericity from 0.82 to 0.95. Alkhatib et al. [33] reported that optimizing the process conditions is essential for obtaining spherical capsules/pearls of an acceptable uniform size. The optimized theoretical equations (OTE) predicted a sphericity of 0.925, which closely matched the experimental results. Finally, Taguchi’s experimental design allowed the identification of key variables in the encapsulation process of *P. nalgiovense* that greatly influenced the formation of spherical capsules.

### 3.3. Morphological Analysis of Alginate–Chitosan Capsules

**Figure 2:**
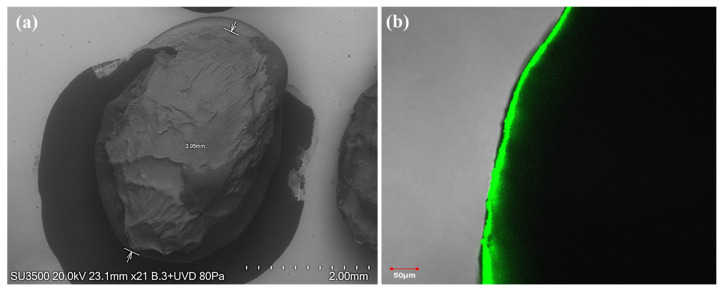
Alginate–chitosan–*P. nalgiovense* capsules microstructure as revealed by (**a**) scanning electron microscopy (SEM) and (**b**) confocal laser scanning microscopy (CLSM), (2.0% alginate solution extruded into an aqueous calcium chloride solution (130 mM) containing 1.0% *w*/*w* chitosan).

SEM observed the morphology of the alginate–chitosan–*P. nalgiovense* capsules were prepared by jet-break extrusion and ionic gelation (Figure 2a). The capsules exhibited spherical shapes but with rough and ridged surfaces, with a particle size of around 4 mm. CLSM analysis showed the fluorescence of chitosan around the capsules (Figure 2b), confirming the presence of this polysaccharide as an encapsulating material.

### 3.4. Viability of Encapsulated Fungus

**Figure 3:**
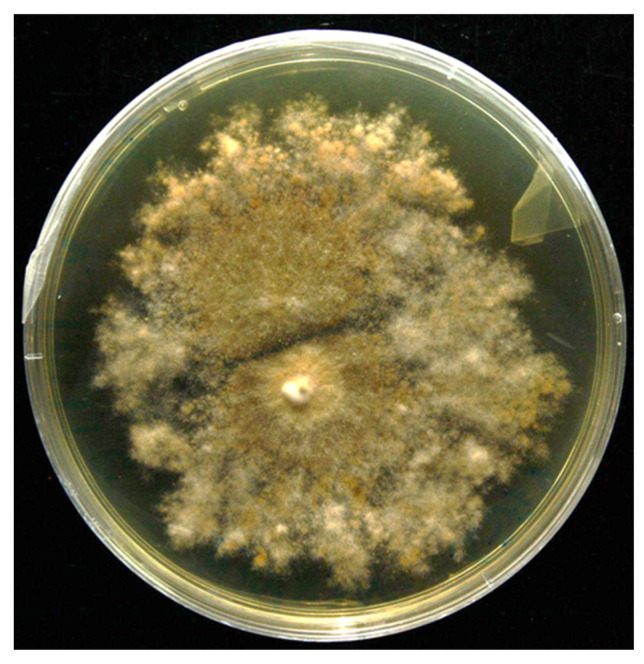
Viability of the encapsulated *P. nalgiovense* following 20 min of disinfection in 1% sodium hypochlorite and subsequent culture on PDA for 7 days at 26 °C.

The results showed that the encapsulated fungus retained viability at all tested disinfection times (2, 5, 10, and 20 min). In addition, *P. nalgiovense* showed consistent growth on both media used for viability assessment. The fungal colony displayed radial expansion on PDA and its characteristic morphology, indicating active metabolic activity (Figure 3). On PDB, the encapsulated fungus showed robust growth, as evidenced by an increase in biomass during the incubation period.

## 4. Discussion

In arid conditions, such as those in the Atacama Desert, characterized by high temperatures, soil salinity, low rainfall, and nutrient deficiencies, plant growth-promoting microorganisms, including endophytes, have gained attention for their ability to effectively colonize roots, mobilize soil minerals, and mitigate biotic and abiotic stresses [34,35]. The endophytic fungi from arid environments, such as *P. nalgiovense*, play a crucial role in improving plant resilience. The identified PGP traits suggest that this fungus enhances nutrient uptake by actively participating in nitrogen fixation and improving the bioavailability of minerals such as phosphorus and iron under harsh conditions. In addition, the production of EPS can alleviate abiotic stress, as EPS are hydrated compounds that help fungi protect themselves and plant roots against desiccation while also improving soil structure and aggregate stability [36]. In this context, the use of stress-adaptive microorganisms with high PGP activity serves as a valuable bioresource, showing significant potential for the development of encapsulated bioinoculants to manage abiotic stress.

Encapsulation by syringe with ionic gelation allows the production of small quantities of capsules but is limited by the low reproducibility of manual preparation [12,37]. Spherical capsules or beads have been reported to offer improved stability compared to irregular or non-spherical shapes in encapsulation systems due to a lower surface-to-volume ratio, which minimizes diffusion of the material to be protected [21,38]. Therefore, this study developed a more precise approach to address this limitation and improve the production of spherical and uniform capsules containing the *P. nalgiovense* fungus with high reproducibility. For this purpose, two encapsulation techniques were used: ionic gelation and annular jet burst extrusion, achieving spherical alginate–chitosan–*P. nalgiovense* capsules. The results showed that the content of chitosan as encapsulating material of the capsules with *P. nalgiovense* was the most influential variable on the sphericity when the percentage of chitosan was 1%. One study revealed that applying chitosan as an encapsulating material at low concentrations can produce physically stable calcium alginate capsules. This encapsulating material also mitigates the destructive effects of calcium ion-chelating agents on the capsule structure [39]. The second most influential variable on sphericity was the distance from the nozzle to the gelation bath (5 cm). One study has reported that the optimal distance from the nozzle to the gelling bath to obtain high sphericity depends on the viscosity of the encapsulating solutions, i.e., the optimal nozzle distance for low viscosity liquids (<80 mPa*s) was less than 19 cm and for high viscosity liquids (>190 mPa*s) was 35–135 cm [40]. In our study, the viscosities of alginate (2%) and chitosan (1%) solutions were lower than 30 mPa*s, which agrees with the results reported by the aforementioned authors. Finally, the vibration frequency (40 Hz) was the variable with the least influence on sphericity. This variable had more influence on the particle size than on the sphericity of the capsules. It has been reported that capsule size is inversely proportional to frequency, i.e., as frequency increases, bead and/or capsule size decrease [12]. Finally, these three process variables obtained spherical alginate–chitosan–*P. nalgiovense* capsules using jet-breaking extrusion encapsulation technology.

The capsules exhibited spherical shapes but with rough and ridged surfaces, probably due to slight shrinkage after dehydration during refrigerated storage. This wrinkling phenomenon is attributed to the partial collapse of the alginate/chitosan hydrogel polymeric alginate/chitosan network during dehydration [41]. Furthermore, morphological analysis corroborated that the alginate–chitosan–*P. nalgiovense* capsules had particle sizes around 4 mm. On the other hand, confocal laser scanning microscopy analysis showed the outer layer of chitosan, visualizing the coating material of the capsules with a thickness of less than 50 µm. A similar behavior was reported by Morales et al. [32], who observed by confocal laser scanning microscopy the fluorescence of the chitosan around the pectin particle, which confirmed the presence of the encapsulating material.

The viability tests demonstrated the protective function of the encapsulation system, ensuring fungal survival under disinfection conditions. The ability of *P. nalgiovense* to persist and remain metabolically active further supports its potential as a bioinoculant. Similar findings have been reported for encapsulated microbial formulations enhancing plant resilience in extreme environments [42].

This study presents a novel encapsulation strategy employing jet-breaking extrusion technology to encapsulate extremophilic root-associated fungi, marking a significant advancement in the development of bioinoculants. The results demonstrate the potential of this approach to produce robust, controlled-release fungal formulations capable of enhancing plant adaptation to arid and metal-contaminated environments.

## 5. Conclusions

The results of this work demonstrate that the endosphere of the Atacama Desert plant *N. chilensis* represents an ecosystem that harbors extremophilic microorganisms with multiple PGP, including ammonia production, EPS synthesis, siderophore and auxin production, and phosphate solubilization. Additionally, this study successfully optimized the *P. nalgiovense* encapsulation process in alginate–chitosan capsules by jet-breaking extrusion. Chitosan concentration (1%), nozzle-to-bath distance (5 cm), and vibration frequency (40 Hz) were identified as key variables influencing sphericity, and an optimum sphericity of 0.914 ± 0.002 was achieved. Taguchi’s method effectively determined the optimum conditions with minimal testing. Morphological analysis revealed spherical capsules with rough surfaces and particle sizes of around 4 mm, while confocal laser scanning microscopy confirmed the integrity of the chitosan coating. Finally, the encapsulated *P. nalgiovense* fungus retained high viability across all tested disinfection times on both PDA and PDB media, exhibiting robust growth and active metabolic activity. These results underline the importance of precise parameter control to improve the reproducibility and stability of fungal encapsulation systems. Furthermore, they provide important information to establish a solid basis for the development of encapsulated bioinoculants.

Finally, it is recommended to evaluate the efficacy of encapsulated bioinoculants produced via jet-breaking extrusion technology under field conditions, assess their interactions with different plant species, and explore the scalability of this process for sustainable agricultural applications in extreme environments such as the Atacama Desert.

## Figures and Tables

**Table 1:** Mean size and sphericity of alginate–chitosan–*P. nalgiovense* capsules using the orthogonal matrix L_4_(2^3^).

Design Point	Frequency (Hz)	Distance (cm)	Chitosan (%)	Mean Size (mm)	Sphericity of Capsules
1	40	5	1	4.91 ± 0.013	0.925 ± 0.002
2	40	15	2	3.44 ± 0.002	0.902 ± 0.003
3	120	5	2	3.64 ± 0.070	0.904 ± 0.004
4	120	15	1	2.17 ± 0.001	0.908 ± 0.000

**Table 2:** Screening of plant growth-promoting (PGP) traits of endophytic fungus *P. nalgiovense* isolated from *Neltuma chilensis* roots.

AP ^a^	IAA ^b^	ACC ^c^	SID ^d^	EPS ^e^	P Solubilization ^f^			
					Ca_3_(PO_4_)_2_	AlO_4_P	FeO_4_P 2H_2_O	Phytic Acid
40.47 ± 0.9	0.94 ± 0.1	ND	6.13 ± 0.3	102.02 ± 3.1	2.6 ± 0.0	5.5 ± 0.0	5 ± 0.0	2.2 ± 0.3

ND: Not detected. ^a^ Ammonia production (µmol mL^−1^). ^b^ Acid indoleacetic production (µmol mL^−1^). ^c^ ACC activity (µmol α—KT mg^−1^ protein h^−1^). ^d^ Siderophore production (percent siderophore unit, PSU). ^e^ Exopolysaccharide production (mg L^−1^). ^f^ P source solubilization (phosphate solubilization index, PSI). Values are expressed as means ± standard error with n = 5.

## Data Availability

The original contributions presented in the study are included in the article, further inquiries can be directed to the corresponding author.
